# Identification of Virulence Factors Genes in *Escherichia coli* Isolates from Women with Urinary Tract Infection in Mexico

**DOI:** 10.1155/2014/959206

**Published:** 2014-05-05

**Authors:** Daniela A. López-Banda, Erika M. Carrillo-Casas, Margarita Leyva-Leyva, Gabriel Orozco-Hoyuela, Ángel H. Manjarrez-Hernández, Sara Arroyo-Escalante, David Moncada-Barrón, Silvia Villanueva-Recillas, Juan Xicohtencatl-Cortes, Rigoberto Hernández-Castro

**Affiliations:** ^1^Department of Ecology of Pathogen Agents, Hospital General “Dr. Manuel Gea González”, 14080 Tlalpan, DF, Mexico; ^2^Department of Molecular Biology and Histocompatibility, Hospital General “Dr. Manuel Gea González”, 14080 Tlalpan, DF, Mexico; ^3^Institute of Cell Physiology, Universidad Nacional Autónoma de México, 04510 Coyoacán, DF, Mexico; ^4^Department of Public Health, Universidad Nacional Autónoma de México, 04510 Coyoacán, DF, Mexico; ^5^Clinical Laboratory, Hospital General “Dr. Manuel Gea González”, 14080 Tlalpan, DF, Mexico; ^6^Department of Infectology, Hospital Infantil de México “Federico Gómez”, 06720 Cuauhtémoc, DF, Mexico

## Abstract

*E coli* isolates (108) from Mexican women, clinically diagnosed with urinary tract infection, were screened to identify virulence genes, phylogenetic groups, and antibiotic resistance. Isolates were identified by MicroScan4 system; additionally, the minimum inhibitory concentration (MIC) was assessed. The phylogenetic groups and 16 virulence genes encoding adhesins, toxins, siderophores, lipopolysaccharide (LPS), and invasins were identified by PCR. Phylogenetic groups distribution was as follows: B1 9.3%, A 30.6%, B2 55.6%, and D 4.6%. Virulence genes prevalence was *ecp* 98.1%, *fimH* 86.1%, *traT* 77.8%, *sfa/focDE* 74.1%, *papC* 62%, *iutA* 48.1%, *fyuA* 44.4%, *focG* 2.8%, *sfaS* 1.9%, *hlyA* 7.4%, *cnf-*1 6.5%, *cdt-B* 0.9%, *cvaC* 2.8%, *ibeA* 2.8%, and *rfc* 0.9%. Regarding antimicrobial resistance it was above 50% to ampicillin/sulbactam, ampicillin, piperacillin, trimethoprim/sulfamethoxazole, ciprofloxacin, and levofloxacin. Uropathogenic *E. coli* clustered mainly in the pathogenic phylogenetic group B2. The isolates showed a high presence of siderophores and adhesion genes and a low presence of genes encoding toxins. The high frequency of *papC* gene suggests that these isolates have the ability to colonize the kidneys. High resistance to drugs considered as first choice treatment such as trimethoprim/sulfamethoxazole and fluoroquinolones was consistently observed.

## 1. Introduction

Urinary tract infections (UTI) are one of the most common infections worldwide. Uropathogenic* Escherichia coli* (UPEC) is the primary pathogen causing UTIs; it colonizes the human intestine a few hours after birth and is considered part of the normal microbiota. However, it can cause various diseases such as diarrhea, UTI, and meningitis [1]. It is classified into three groups: (i) commensal, (ii) intestinal pathogenic, and (iii) extraintestinal pathogenic [2], and phylogenetically it has been classified into four classic groups (A, B1, B2, and D) [3]. Uropathogenic* E. coli* is located within the extraintestinal pathogenic* E. coli *(ExPEC), classified primarily into the phylogenetic group B2 and to a lesser extent to group D, whereas commensal strains are within the phylogenetic groups A and B1 [4–8].

The ability of* E. coli* to colonize different anatomical sites is due in part to genome plasticity and remodeling by acquisition or loss of genetic material from which it acquired resistance or virulence factors. Therefore, horizontal transfer is an important factor in the evolution and adaptation of* E. coli* to different niches [9, 10]. The interaction between bacteria and epithelial cells is a multifactorial and complex phenomenon which involves several adhesins produced according to the stage of infection, while adherence to epithelial cells is essential for successful colonization and establishment; the expression of other genes encoding toxins, siderophores, lipopolysaccharide (LPS), capsule, and invasins determines the disease severity and the strain's virulence [8]. UPEC strains can cause acute infections and recurrent infections that do not respond to common antimicrobial treatments. UTI treatment generally includes *β*-lactam antibiotics, fluoroquinolones, or trimethoprim/sulfamethoxazole [11–13] but may vary according to patient age, sex, Pathogen involved, course of disease, and the urinary tract anatomic area involved [5]. The increased resistance may be related to changes in the bacterial genome by mutation or acquisition by horizontal transfer of an extrachromosomal or chromosomal material [14–16].

Urinary tract successful invasion depends on the bacteria virulence, inoculums size, and the host's defense mechanisms [18]. However, women have higher UTI's prevalence and incidence mainly due to their anatomical characteristics such as the proximity between the anus and the urethral opening, hormone effects, and changes in the genital microbiota [14, 19]. Clinically a UTI is defined by a bacteriuria with a count in midstream urine culture ≥10^5^ CFU/mL and pyuria or the presence of white blood cells in the urine, more than five leukocytes per field [19].

Globally it is estimated that about 150 million UTIs occur annually [20]. In the United States and Spain the current situation and treatment of urinary tract infections had been thoroughly described [6–8, 18]; it is estimated that 11% of women experience at least one diagnosis of urinary tract infection (UTI) per year, and 60% of women will have or have had an UTI or more during their lifetime [4]. In Mexico UTI's status has not been described. However, to our knowledge it is* E. coli* one of the pathogenic agents of UTI and it is more frequent in women, with high incidence and prevalence, representing a costly problem for the health sector [4, 21]. In 2008, 3,244,994 cases were reported, which represents an incidence of 3,041.7/100,000 inhabitants, from which 75.6% (2,453,608/100,000 inhabitants) were women, representing an incidence of 4,508.6/100,000.

The present study aimed to describe the profile of* E. coli* from Mexican women with urinary tract infection by the identification of virulence genes* (fimH, papC, sfa/focDE, sfaS, focG, ecpA, ecpR-B, hlyA, cnf-1, cdt-B, cvaC, iutA, ibeA, rfc, tratT, *and* fyuA)*, phylogenetic group, and their resistance to antibiotics to guide better diagnosis and treatment of UTI.

## 2. Material and Method

### 2.1. Bacteria and Culture

Bacterial isolates (108) were obtained from urine samples from women diagnosed with acute urinary tract infection and confirmed by the clinical laboratory of the General Hospital “Dr. Manuel Gea Gonzalez” during 2008 and until 2010. All samples with counts over 100,000 UFC/mL were included. Patients were within an age range between 12 and 58 years and mean age of 38.9 years. Isolates were identified by MicroScan 4 (Dade Behring) automated system. The presence of *β*-lactamases and the minimum inhibitory concentration (MIC) were also determined for ampicillin, ampicillin/sulbactam, amoxicillin/clavulanic acid, aztreonam, imipenem, meropenem, piperacillin, piperacillin/tazobactam, ticarcillin/clavulanic acid, amikacin, gentamicin, tobramycin, ceftriaxone, ceftazidime, cefotaxime, cefoxitin, cephalotin, cefazolin, cefepime, cefuroxime, cefotetan, trimethoprim/sulfamethoxazole, ciprofloxacin, gatifloxacin, levofloxacin, and moxifloxacin. The antibiotic resistance was classified into sensitive, resistant, and ESBL (resistance due to *β*-lactamases). Pure cultures were maintained at −70°C in brain-heart infusion broth/glycerol 50%. The* E. coli* CFT073 uropathogenic strain was used as control strain.

### 2.2. Phylogenetic Groups and Virulence Factors

The PCR was performed with the GoTaq Flexi kit (Promega) according to the manufacturer's instructions. Phylogenetic groups were identified according to Clermont protocol [22]. 16 virulence genes of UPEC were included:* fimH, papC, sfa/focDE, sfaS, focG, ecpA, ecpR-B, hlyA, cnf-1, cdt-B, cvaC, iutA, ibeA, rfc, tratT, *and* fyuA*. PCR primers and conditions for each gene are described in Tables 1 and 2 [5, 17]. The* ecp RB* PCR was performed to overcome the possible variation of* ecpA *which may give a false negative result of the* E*.* coli* common pilus [17]. All PCR products were visualized in agarose gel stained with ethidium bromide.

### 2.3. Statistical Analysis

To establish the results significance, the Fisher exact test was used. The level of significance was set at a* P* value of ≤0.05.

## 3. Results

The overall results of the isolates regarding the virulence genes, the phylogenetic group, and resistance profile are shown in Table 3. Regarding the phylogenetic group, most of the isolates were (60) grouped into the B2 group (55.6%), 33 isolates were classified as part of the A group (30.6%), 10 isolates (9.3%) to group B1, and 5 isolates (4.6%) to group D.

### 3.1. Virulence Genes

Higher prevalence, above 50%, was observed for the* ecp*,* fimH*,* traT*,* sfa*/*focDE,* and* papC* genes (98.1%, 86.1%, 77.8%, 74.1%, and 62%, resp.). For* iutA* and* fyuA *genes prevalence was close to 50% (48.1% and 44.4%, resp.), while the* focG*,* sfaS*,* hlyA*,* cnf*-1,* cdt*-*B*,* cvaC*,* ibeA*, and* rfc *genesregistered prevalence lower than 10% (2.8%, 1.9%, 7.4%, 6.5%, 0.9%, 2.8%, 2.8%, and 0.9%, resp.).  Table 4 shows the distribution of virulence genes regarding the phylogenetic group. Most of the virulence factors associated with the phylogenetic group B2 were identified. The* ecp *(*A and R-B*) and* fimH* genes are widely distributed among all groups (A 100%/78.8%, B1 100%/70%, B2 96.7%/91.7%, and D 100%/100%, resp.). The* focG, sfaS, hlyA, cnf-1, cdt-B, *and* cvaC* genes were found only in isolates from the B2 group. The* rfc *gene was found in just one isolate from group B1. The* hlyA, cft-1,* and* traT *genes were positively associated with group B2, and the* iutA* and* fyuA *genes were negatively associated with group A.

### 3.2. Antibiotic Resistance

Above 50% of antibiotic resistance was observed for ampicillin/sulbactam (75.9%), ampicillin (55.2%), piperacillin (51.1%), trimethoprim/sulfamethoxazole (56.1%), ciprofloxacin (62.3%), gatifloxacin (62.5%), levofloxacin (60.2%), and moxifloxacin (52.6%). Sensitivity values above 50% were found to amoxicillin/clavulanic acid (68.8%), aztreonam (78.4%), imipenem (98.1%), meropenem (100%), piperacillin/tazobactam (86%), ticarcillin/clavulanic acid (58.2%), amikacin (93.5%), gentamicin (72.2%), tobramycin (56.5%), ceftriaxone (78.1%), ceftazidime (77.9%), cefotaxime (78.9%), cefoxitin (91.1%), cefazolin (65.9%), cefepime (78.1%), cefuroxime (71.1%), and cefotetan (98.4%). Approximately 20% of isolates registered the presence of *β*-lactamases and around 20% were resistant to antimicrobials, as shown in Table 5. Isolates which displayed resistance to more than ≥3 chemotherapeutic groups were considered multiresistant isolates, which represents 58%. However no statistical relation was observed among multiresistance and phylogenetic group (Table 6).

## 4. Discussion

In this work 108* E. coli* isolates were screened from female patients with an average age of 39 years; women were regarded as a productive population, for which urinary tract infections are considered a major cause of morbidity in our country and represent a huge economic impact [23, 24].

The predominant phylogenetic group was B2 (55.6%), widely associated with pathogenic strains. In Spain and the United States similar results had been reported and also a lower percentage related to the phylogenetic group D [6–8, 18, 25]. The phylogenetic group A, associated with commensal strains, represents a 30.6%, higher than in other studies, suggesting that the gastrointestinal tract is the main reservoir of strains that may be able to colonize the urinary tract in accordance to previous observations [6, 7, 18, 25]. The B1 group (9.3%) as a cause of urinary tract infections points out the high plasticity of the* E coli* genome which allowed the presence of the* fimH, papC, and ecp* (*A* and* RB*) in percentages of 70%, 60%, and 100%, respectively. These genes are related to the ability to colonize the urinary tract epithelium [18, 22, 26–28].

Adhesins genes were present in high percentages:* fimH *(86.1%),* ecp *(*A y R-B*) (98.1%), and* papC *(62%), this result could be related to the pathogenicity of the isolated strains as adherence is the most important pathogenicity determinant [4]. The* fimH* geneonce again was highly conserved in UTI isolates which confirms its crucial role during colonization of the urinary tract [4, 29–32].

The* ecp *(*A *and* RB*) gene is associated with commensals and enteropathogenic strains; it was present in 98.1% of this study isolates and according to a similar observation in Portugal it was found in 100% of their isolates; it may be associated with UPEC [17, 22, 24, 28]. The* papC* gene encodes an outer membrane protein essential for the fimbriae P biogenesis regulation.* pap* genes presence had been associated with pyelonephritis; therefore, higher percentages (over 50%) suggest that the strains isolated from the Mexican population have greater capabilities to colonize kidneys and generate pyelonephritis [32, 33].

The* hlyA* and* cnf-1* genes showed a positive relationship with the B2 group; also they are associated with pathogenicity island PAI II_J96_, and the* iutA* gene is associated with pathogenicity island PAI I_CFT073_ as well as with* hlyA* and* pap* operon [8, 19, 34–36].

The* sfaS *gene was found exclusively in* hlyA* and* cnf-1* positive isolates which could be linked to cystitits cases. This observation is in accordance with the previous report by Lloyd et al. [19].

The* cvaC* gene was present in only three isolates* traT* positive. These genes are both located at the* colV* plasmid.* traT* is related to the phylogenetic group B2, and presumes an animal source. The* iutA* and* fyuA *genesalso showed a relation with the phylogenetic group B2 [37–39]. The* ibeA *gene, related to the B2 group, was found in an isolate identified as B1, a result that may start to change the previous assumption [40]. The* rfc *gene was identified in just one isolate which indicates that the serogroup O4 was not the predominant serogroup in the population studied and that this result may need further serological confirmation [5, 41].

The treatment of choice for ITU is in order of importance: fluoroquinolones (ciprofloxacin), the trimethoprim/sulfamethoxazole, cephalosporins, and penicillins (ampicillin) to which an increasingly developed resistance has been reported due mainly to the indiscriminated antibiotic use [13, 14]. In this work it is confirmed the resistance previously reported values for trimethoprim/sulfamethoxazole (56.1%) [14, 42]; for ciprofloxacin (62.3%), gatifloxacin, levofloxacin, and moxifloxacin, resistance was always above 50%. Positive isolates to* hlyA, cnf-1, *and/or* papC* genes were susceptible to fluoroquinolones, results similar to those of Piatti et al. [43]. Besides in Mexico, the previously reported* E. coli* resistance profile included ampicillin, piperacillin, fluoroquinolones, and trimethoprim/sulfamethoxazole which are considered to be first-line choices [13, 44, 45]. Additionally, serotype 025b-ST131 has been reported to be within the Mexican population which has been associated with plasmid mediated quinolone resistance [46]. We had identified multidrug resistance of the* E. coli* strains causing UTI in 58% of the isolates which belonged mainly to group B2, result which kept our attention.

## 5. Conclusion

This work confirms that most of the isolates associated with urinary tract infections belong to the phylogenetic group B2 and in a lesser extent to group D. Also they displayed a great number of virulence genes. However, commensal strains may also be the cause of UTI. According to our results most parts of the isolates have the ability to colonize the kidneys as they have a high incidence of the* papC* gene. The* hlyA *and* cnf-1* genes encoding toxins and* fyuA iutA* and siderophores encoding genes are tightly associated with the phylogenetic group B2.

*E. coli* has successfully adapted to host's conditions and to the general medical practices as we may observe the high resistance to trimethoprim/sulfamethoxazole and fluoroquinolones, especially on the most frequently isolated phylogenetic groups.

Finally, these results reinforce international knowledge on antimicrobial resistance and the high rate of multidrug resistance found invites us to encourage population awareness of the proper use of antimicrobials.

## Figures and Tables

**Table 1:** PCR primer for each virulence factor. Primer sequence was taken from Johnson and Stell, 2000 [5] and Blackburn et al., 2009 [17].

Genes	Primer (5′-3′)

*ecpA *	TGA AAA AAA AGG TTC TGG CAA TAG C
	CGC TGA TGA GGA GAA AGT GAA

*ecpRB *	GTC ACA TGG CAA AAT GAT TAC AGC
	TCA CGG GAA TGA ACT TAT CAC CC

*papC *	GTG GCA GTA TGA GTA ATG ACC GTT A
	ATA TCC TTT CTG CAG GGA TGC AAT A

*sfaS *	GTG GAT ACG ACG ATT ACT GTG
	CCG CCA GCA TTC CCT GTA TTC

*focG *	CAG CAC AGG CAG TGG ATA CGA
	GAA TGT CGC CTG CCC ATT GTC

*fimH *	TGC AGA ACG GAT AAG CCG TGG
	GCA GTC ACC TGC CCT CCG GTA

*sfa/fogDE *	CTC CGG AGA ACT GGG TGC ATC TTA C
	CGG AGG AGT AAT TAC AAA CCT GGC A

*cnf1 *	AAG ATG GAG TTT CCT ATG CAG GAG
	CAT TCA GAG TCC TGC CCT CAT TAT T

*hlyA *	AAC AAG GAT AAG CAC TGT TCT GGC T
	ACC ATA TAA GCG GTC ATT CCC GTC A

*cdt-s *	GAA AGT AAA TGG AAT ATA AAT GTC CG
	GAA AAT AAA TGG AAC ACA CAT GTC CG

*cdt-a *	AAA TCA CCA AGA ATC ATC CAG TTA
	AAA TCT CCT GCA ATC ATC CAG TTT A

*colV *	CAC ACA CAA ACG GGA GCT GTT
	CTT CCC GCA GCA TAG TTC CAT

*fyuA *	TGA TTA ACC CCG CGA CGG GAA
	CGC AGT AGG CAC GAT GTT GTA

*iutA *	GGC TGG ACA TCA TGG GAA CTG G
	CGT CGG GAA CGG GTA GAA TCG

*ibeA *	AGG CAG GTG TGC GCC GCG TAC
	TGG TGC TCC GGC AAA CCA TGC

*Rfc *	ATC CAT CAG GAG GGG ACT GGA
	AAC CAT ACC AAC CAA TGC GAG

*traT *	GGT GTG GTG CGA TGA GCA CAG
	CAC GGT TCA GCG ATC CCT GAG

**Table 2:** PCR conditions for each gene.

Genes	Inicial denaturation (°C/min)	Denaturation (°C/s)	Annealing (°C/s)	Extension (°C/s)	Final extention (°C/min)	Cycles
*ecpA *	96/5	94/30	62/45	72/45	72/5	35
*ecpR-B *	95/5	95/30	57.5/33	72/90	75/5	35
*fimH *	96/5	94/30	65.5/30	72/30	72/5	35
*papC *	95/5	94/30	58.2/30	72/40	72/5	40
*sfaS *	95/5	94/30	64/30	72/25	72/5	35
*fogG *	95/5	95/30	64/40	72/30	72/5	30
*sfa/focDE *	95/5	94/30	65/30	68/40	72/3	35
*cnf-1 *	95/5	94/30	65.5/30	72/30	72/5	35
*hlyA *	95/5	94/30	63/30	68/60	72/5	30
*Mu* *lt* *i*1*	95/5	94/30	67.1/30	68/160	72/5	30
*Mu* *lt* *i*2**	95/5	94/30	61.5/30	68/180	72/5	35
*Rfc *	95/5	95/30	62.5/30	72/60	75/5	40

*Multiplex 1 for genes fyuA, iutA e ibeA.**Multiplex 2 for genes *cdtB, cvaC y traT.*

**Table 3:** Virulence genes and phylogenetic group.

*# Strain *	*Ecp *	*fimH *	*papC *	*sfa/focDE *	*focG *	*sfaS *	*hlyA *	*cnf-1 *	*cdtB *	*cvaC *	*iutA *	*ibeA *	*Rfc *	*traT *	*fyuA *	*chuA *	*yja *	*TSP *	*PG *
1	+	+	+	+	−	−	−	−	−	+	+	+	−	+	+	+	+	+	B2
2	+	+	−	−	−	−	−	−	−	−	+	−	−	+	+	+	−	−	D
3	+	+	+	−	−	−	−	−	−	−	+	−	−	+	+	+	+	+	B2
4	+	+	+	+	−	−	−	−	−	−	−	−	−	−	−	−	+	−	A
5	+	+	+	+	−	−	−	−	−	−	−	−	−	−	−	−	+	−	A
6	+	+	+	−	−	−	−	−	−	−	+	−	−	+	+	−	+	−	A
7	+	+	+	+	−	−	−	−	−	−	+	−	−	+	+	+	+	−	B2
8	+	−	+	+	−	−	−	−	−	−	+	−	−	+	+	+	+	+	B2
9	+	+	+	+	−	−	−	−	−	−	−	−	−	−	−	−	+	−	A
10	+	+	+	+	−	−	−	−	−	−	−	−	−	+	+	+	+	−	B2
11	+	−	+	+	−	−	−	−	−	−	−	−	−	+	−	−	+	−	A
12	−	+	+	−	−	−	−	−	−	−	+	−	−	+	+	+	+	+	B2
13	+	+	+	+	−	−	−	−	−	−	+	−	−	+	+	+	+	+	B2
14	+	+	+	+	−	−	−	−	−	−	−	−	−	−	−	+	+	−	B2
15	+	−	+	+	−	−	−	−	−	−	+	−	−	+	+	+	+	+	B2
16	+	+	+	+	−	−	−	−	−	−	+	−	−	+	−	−	+	−	A
17	+	+	+	+	−	−	−	−	−	−	+	−	−	+	−	−	+	−	A
18	+	+	+	+	−	−	−	−	−	−	+	−	−	+	+	+	+	−	B2
19	+	+	−	+	−	−	−	−	−	−	+	−	−	+	+	+	+	+	B2
20	+	+	+	+	−	−	−	−	−	−	−	−	−	+	−	−	+	+	B1
21	+	+	+	+	−	−	−	−	−	−	+	−	−	+	+	+	+	−	B2
22	+	+	+	+	−	−	−	−	−	−	−	−	−	+	−	+	+	+	B2
23	+	+	+	+	−	−	−	−	−	−	+	−	−	+	+	−	−	−	A
24	+	+	+	+	−	−	−	−	−	−	−	−	−	+	−	+	+	+	B2
25	+	+	+	+	−	−	−	−	−	−	−	−	−	−	−	−	+	−	A
26	−	+	+	+	−	−	−	−	−	−	−	−	−	+	−	+	+	−	B2
27	+	+	+	+	−	−	−	−	−	−	−	−	−	+	−	+	+	−	B2
28	+	+	−	+	−	−	−	−	−	−	+	−	−	+	+	+	+	+	B2
29	+	+	−	+	−	−	−	−	−	−	−	−	−	−	−	+	−	−	D
30	+	+	+	+	+	−	+	+	−	−	−	−	−	+	+	+	+	+	B2
31	+	+	−	+	−	−	−	−	−	−	+	−	−	+	+	−	+	+	B1
32	+	+	+	+	−	−	+	+	−	−	−	−	−	+	−	+	+	+	B2
33	+	+	−	+	−	−	−	−	−	−	−	−	−	−	−	+	+	−	B2
34	+	+	+	−	−	−	−	−	−	−	−	−	−	+	+	−	+	−	A
35	+	+	−	+	−	−	−	−	−	−	+	−	−	−	+	+	+	+	B2
36	+	+	−	+	−	−	−	−	−	−	−	−	−	+	−	+	−	−	D
37	+	+	+	−	−	−	−	−	−	−	+	−	−	+	+	+	+	+	B2
38	+	+	+	−	−	−	−	−	−	−	−	−	−	+	−	+	+	+	B2
39	+	+	+	+	−	−	−	−	−	−	+	−	−	+	−	+	−	−	D
40	+	+	+	+	−	−	−	−	−	−	−	−	−	+	−	+	+	−	B2
41	+	+	+	−	−	−	−	−	−	−	+	−	−	+	+	+	+	+	B2
42	+	+	−	−	−	−	−	−	−	−	+	−	−	+	+	+	+	+	B2
43	+	+	−	−	−	−	−	−	−	−	−	−	−	+	+	−	−	−	A
44	+	+	−	−	−	−	−	−	−	−	−	−	−	−	−	−	+	−	A
45	+	+	+	+	−	−	−	−	−	−	−	−	−	−	−	−	+	−	A
46	+	+	+	+	−	−	−	−	−	−	+	−	−	+	+	+	+	−	B2
47	+	+	+	+	−	−	−	−	−	−	+	−	−	+	+	+	+	−	B2
48	+	+	+	+	−	−	−	−	−	−	−	−	−	−	−	−	+	−	A
49	+	+	−	+	−	−	−	−	−	−	−	−	−	+	+	+	+	−	B2
50	+	+	−	+	−	−	−	−	−	−	−	−	−	+	+	−	+	−	A
51	+	+	+	+	−	−	−	−	−	−	−	−	−	−	−	+	+	+	B2
52	+	+	+	+	−	−	−	−	−	−	−	−	−	−	−	−	+	−	A
53	+	+	−	−	−	−	−	−	−	−	−	−	−	−	−	−	+	−	A
54	+	+	+	−	−	−	−	−	−	−	−	−	−	+	+	+	+	−	B2
55	+	+	+	+	+	−	−	−	−	+	+	−	−	+	−	+	+	−	B2
56	+	+	+	+	−	−	−	−	−	−	+	−	−	+	−	−	+	−	A
57	+	+	+	−	−	−	−	−	−	−	+	−	−	+	+	+	+	+	B2
58	+	−	+	−	−	−	−	−	−	−	−	−	−	−	−	−	−	+	B1
59	+	+	+	+	−	−	−	−	−	−	−	−	−	−	−	+	−	+	D
60	+	−	+	−	−	−	−	−	−	−	+	−	−	−	−	−	+	−	A
61	+	+	−	+	−	−	−	−	−	−	−	−	−	−	−	−	−	−	A
62	+	+	−	−	−	−	−	−	−	−	+	−	−	+	+	+	+	+	B2
63	+	+	+	+	−	−	−	−	−	−	+	−	−	+	+	+	+	+	B2
64	+	+	−	−	−	−	−	−	−	−	+	−	−	+	+	+	+	+	B2
65	+	+	+	−	−	−	−	−	−	−	+	−	−	−	+	+	+	+	B2
66	+	+	+	+	+	−	+	+	−	−	−	−	−	+	−	+	+	+	B2
67	+	+	+	−	−	−	−	−	−	−	−	−	+	−	−	−	+	+	B1
68	+	+	−	+	−	−	−	−	−	−	−	−	−	+	−	+	+	+	B2
69	+	+	+	+	−	−	−	−	−	−	−	−	−	+	−	+	+	−	B2
70	+	+	−	−	−	−	−	−	−	−	−	−	−	+	+	−	+	−	A
71	+	+	+	+	−	−	−	−	−	−	+	−	−	+	−	−	+	−	A
72	+	+	−	+	−	−	−	−	−	−	−	−	−	−	−	−	−	−	A
73	+	+	−	+	−	−	−	−	−	−	−	−	−	+	−	−	+		A
74	+	+	+	+	−	+	+	+	−	−	−	−	−	+	+	+	+	+	B2
75	+	+	−	−	−	−	−	−	−	−	+	−	−	+	+	+	+	−	B2
76	+	−	−	−	−	−	−	−	−	−	−	−	−	+	−	−	+	−	A
77	+	+	−	+	−	−	−	−	−	−	−	−	−	+	+	+	+	−	B2
78	+	+	−	−	−	−	−	−	−	−	−	−	−	+	−	−	+	−	A
79	+	+	−	+	−	−	−	−	−	−	+	−	−	−	−	+	+	−	B2
80	+	+	+	+	−	−	−	−	−	−	−	−	−	−	−	+	+	−	B2
81	+	+	+	+	−	−	−	−	−	−	+	−	−	+	+	+	+	+	B2
82	+	+	−	+	−	−	−	−	−	−	−	−	−	+	+	−	+	−	A
83	+	+	+	+	−	+	+	+	−	−	+	−	−	+	+	+	+	+	B2
84	+	−	+	+	−	−	−	−	−	−	+	−	−	+	−	−	+	−	A
85	+	−	+	+	−	−	−	−	−	−	−	−	−	+	−	−	+	−	A
86	+	+	+	−	−	−	−	−	−	−	+	−	−	+	+	+	+	+	B2
87	+	+	−	−	−	−	−	−	−	−	+	−	−	+	−	−	+	−	A
88	+	+	+	−	−	−	−	−	−	−	−	−	−	+	−	−	+	−	A
89	+	+	−	+	−	−	−	−	−	−	−	−	−	+	+	−	+	+	B1
90	+	+	+	+	−	−	−	−	−	−	+	−	−	+	−	−	+	+	B1
91	+	−	−	+	−	−	−	−	−	−	−	−	−	−	−	−	+	+	B1
92	+	−	+	+	−	−	−	−	−	−	−	−	−	+	−	−	+	+	B1
93	+	+	+	+	−	−	−	−	−	−	−	−	−	+	−	−	+	+	B1
94	+	+	+	+	−	−	−	+	−	−	+	−	−	+	+	+	+	−	B2
95	+	+	+	+	−	−	−	−	−	−	+	−	−	+	+	+	+	+	B2
96	+	+	+	+	−	−	+	−	−	−	+	−	−	+	+	+	+	+	B2
97	+	−	−	+	−	−	−	−	−	−	−	−	−	+	−	−	+	−	A
98	+	+	+	+	−	−	+	+	+	−	+	−	−	+	+	+	+	+	B2
99	+	+	−	+	−	−	−	−	−	−	−	−	−	+	−	+	+	+	B2
100	+	+	−	+	−	−	−	−	−	−	+	−	−	+	+	+	+	+	B2
101	+	+	−	+	−	−	−	−	−	−	+	−	−	+	+	+	+	+	B2
102	+	+	−	−	−	−	−	−	−	−	+	+	−	+	−	−	+	+	B1
103	+	−	−	+	−	−	−	−	−	−	+	−	−	+	−	+	+	−	B2
104	+	−	−	+	−	−	−	−	−	+	+	+	−	+	−	+	+	+	B2
105	+	+	−	+	−	−	+	−	−	−	+	−	−	+	−	+	+	+	B2
106	+	+	−	+	−	−	−	−	−	−	+	−	−	+	−	+	+	+	B2
107	+	−	−	+	−	−	−	−	−	−	−	−	−	+	−	−	+	−	A
108	+	−	−	+	−	−	−	−	−	−	−	−	−	+	−	+	+	+	B2

Phylogenetic group (PG).

**Table 4:** Relation among phylogenetic group and virulence genes.

Gene	Phylogenetic group (*n*, %)			
	A (*n* = 33)	B1 (*n* = 10)	B2 (*n* = 60)	D (*n* = 5)
*Ecp *	33 (100)	10 (100)	58 (96.7)	5 (100)
*fimH *	26 (78.8)	7 (70)	55 (91.7)	5 (100)
*papC *	19 (57.6)	6 (60)	40 (66.7)	2 (40)
*sfa/focDE *	22 (66.7)	7 (60)	47 (78.3)	4 (80)
*focG *	0	0	3 (5)	0
*sfaS *	0	0	2 (3.3)	0
*hlyA *	0	0	8 (13.3)^a^	0
*cnf-1 *	0	0	7 (11.7)^a^	0
*cdt-B *	0	0	1 (1.7)	0
*cvaC *	0	0	3 (5)	0
*iutA *	9 (27.3)^(a)^	3 (30)	38 (63.3)^a^	2 (40)
*ibeA *	0	1 (10)	2 (3.3)	0
*Rfc *	0	1 (10)	0	0
*traT *	21 (63.6)	7 (70)	53 (88.3)^a^	3 (60)
*fyuA *	7 (21.2)^(a)^	2 (20)	38 (63.3)^a^	1 (20)

^a^ *P* values were calculated by comparison of each group with Fisher's exact test.

Statistic significance of ≤0.05^(a)^ negative association.

**Table 5:** Antibiotic resistance.

Antibiotic (number of isolates)	*S* (%)	*R* (%)	ESBL (%)
ampicillin/sulbactam (108)	26 (24.1)	82 (75.9)	0
ampicillin (97)	22 (22.7)	54 (55.7)	21 (21.9)
amoxicillin/clavulinic acid (64)	44 (68.8)	20 (31.3)	0
aztreonam (97)	76 (78.4)	0	21 (21.6)
Imipenem (108)	106 (98.1)	2 (1.9)	0
Meropenem (30)	30 (100)	0	0
piperacillin/tazobactam (107)	92 (86)	15 (14)	0
piperacillin (95)	25 (26.3)	49 (51.6)	21 (22.3)
ticarcillin/clavulanic acid (91)	53 (58.2)	38 (41.8)	0
amikacin (108)	101 (93.5)	7 (6.5)	0
gentamicin (108)	78 (72.2)	30 (27.8)	0
tobramycin (108)	61 (56.5)	47 (43.5)	0
ceftriaxone (96)	75 (78.1)	0	21 (21.9)
ceftazidime (95)	74 (77.9)	0	21 (22.1)
cefotaxime (76)	60 (78.9)	0	16 (21.1)
cefoxitin (45)	41 (91.1)	4 (8.9)	0
cephalotin (22)	10 (45.5)	8 (36.4)	4 (18.2)
cefazolin (91)	60 (65.9)	11 (12.1)	20 (22)
cefepime (96)	75 (78.1)	0	21 (21.9)
cefuroxime (56)	40 (71.4)	1 (1.8)	15 (26.8)
cefotetan (61)	60 (98.4)	1 (1.6)	0
trimethoprim/sulfamethoxazole (107)	47 (43.9)	60 (56.1)	0
ciprofloxacin (106)	40 (37.7)	66 (62.3)	0
gatifloxacin (64)	24 (37.5)	40 (62.5)	0
levofloxacin (108)	43 (39.8)	65 (60.2)	0
moxifloxacin (19)	9 (47.4)	10 (52.6)	0

**Table 6:** Relation among multidrug resistance and phylogenetic group.

Phylogenetic group	Multidrug resistance [positive isolates number (%)]	
	No-MDS (*n* = 45)	MDS (*n* = 63)
A	11 (24.4)	22 (34.9)
B1	6 (13.3)	4 (6.4)
B2	24 (53.3)	36 (57.1)
D	4 (9)	1 (1.6)

MDS: multidrug sensitive. *P* values were calculated by the Fisher's exact test for each group, none has statistical significance value ≤0.05.
